# Editorial: The role of DNA viruses in human cancers

**DOI:** 10.3389/fcimb.2022.1103505

**Published:** 2022-12-08

**Authors:** Ming Hu, Bin Wang, Chengjun Wu

**Affiliations:** ^1^ Department of Special Medicine, Basic Medicine College, Qingdao University, Qingdao, China; ^2^ School of Biomedical Engineering, Dalian University of Technology, Dalian, China

**Keywords:** DNA virus, cancer, prognosis, oncolytic virus, therapy

There is a wide variety of human tumor viruses, including small DNA viruses such as Merkel cell polyomavirus (MCPyV), hepatitis B virus (HBV) and human papillomaviruses (HPV), or large DNA viruses as Kaposi’s sarcoma-associated herpesvirus (KSHV) and Epstein–Barr virus (EBV) (Chen et al., 2021). Around 85% of virus-induced cancers occur in developing countries, which has important implications for the translation of knowledge into public health treatment. As well, certain viruses are likely to contribute to sex-specific differences in tumorigenesis. Almost 90% of HPV-induced cancers are almost exclusively diagnosed in women, whereas HBV, HCV and EBV cancers are generally diagnosed in men (Plummer et al., 2016).

Oncoviruses also differ widely in their carcinogenic mechanisms. Cancers caused by viruses usually arise from chronic infections after many years, which indicates that the infection is just one step among many. Viral gene products regulate anti-apoptotic, proliferative and/or immune escape activities by interacting with cellular genes. Examples of continued expression of specific viral oncoproteins include LMP1 of EBV, E6 and E7 of HPVs and Tax of HTLV-1.

The HPV normally infects stratified epithelium and can cause a variety of anogenital carcinomas, including cervical, vaginal and anal and other mucosal carcinomas including oropharyngeal carcinomas (de Martel et al., 2017). Within two years, the host immune system will be able to clear the majority of HPV infections. In some rare cases, the high-risk HPV infection may develop into persistent infection and cause pre-cancerous lesions to the cervix, which may progress to malignant cervical cancer. HPV gains access to basal layer cells by interacting with heparan sulfate proteoglycans on the cell surface. Although endocytosis is necessary for virus entry into cytoplasm, the cellular entry mechanism is not thoroughly studied. Once infection occurs, HPV genome starts to replicate. In the early stage, the viral genome replicates at a low-level. Baedyananda et al. review summarized that the viral early protein E1 and E2 cooperate to enhance the affinity of viral genome binds to the host cellular DNA replication machinery. During this stage, viral oncoprotein E6 and E7 are generated, the high-risk E6 oncoprotein degrades the tumor suppressor protein p53 resulting in the inhibition of cell apoptosis (McBride, 2017). In an overview, Zheng et al. discussed how several splicing factors control the splicing of high-risk HPV E6/E7 mRNA. E7 binds to tumor suppressor retinoblastoma-associate protein (pRB) and blocks the binding between E7 and its partner protein E2F activating the expression of DNA replication factor. Since the high-risk oncoprotein E6 and E7 are derived from same pre-mRNAs, therefore, the balance of E6 and E7 are vital to the cancer progression. The post-transcriptional modification especially the alternative splicing decides the ratio of E6 and E7. As known, four splice sites locate in HPV16 E6 and E7 coding region including one splicing donor (SD226) and splicing acceptors (SA409, SA526, and SA742) (Ajiro et al., 2012). The unspliced mRNA maintain the entire E6 coding region, it is therefore used for E6 production, while the pre-mRNAs spliced from SD226 to SA409 are used for E7 production. If the splicing is repressed or inhibited, the unspliced E6 level will increase at the expense of E7 level, insufficient E7 cannot completely reduce the pRB, so the infected cells will eventually go to apoptosis. On the contrary, if the splicing is becoming too efficient, the expression level of E7 increases, E6 is too less to completely destroy the apoptosis pathway mediated by p53 thereby shutting down the process of cell carcinogenesis (Olmedo-Nieva et al., 2018 and Cui et al., 2022). It is worth mentioning, besides hnRNPs earlier reported cellular factors including epidermal growth factor (EGF), 5’ cap-binding factors, SRSF1/SRSF2, CTCF, and SF3B1 affect E6/E7 mRNA level further interrupt E6 and E7 protein ratio. In this topic, Lu et al. summarized the clinical features of 19 patients with cervical small cell carcinoma trying to explore more effective therapy for cervical SCNEC. Cui et al. identified five differentially expressed genes in HPV infection related cervical cancer using bioinformatics, which may be used as potential biomarker for cervical screening.

Over 50% of HCC cases are caused by HBV infections worldwide, making them the most significant carcinogenic factor of HCC. There are several factors that can increase the risk of HCC in HBV patients, including gender, age, alcohol consumption, exposure to carcinogens, and duration of infection. While HBV-encoded proteins may directly induce hepato-carcinogenesis, they may also cause cancer indirectly through chronic inflammation and tissue damage caused by persistent infection. Our Research Topic brings together articles that explore the mechanism of HBV infection in HCC, as well as treating HBV-related HCC. In a comprehensive analysis, Li et al. explored a microtubule nucleation factor named TPX2 as a new prognostic biomarker in HBV-related HCC. Specifically, this article identified 541 differential expressed lncRNAs from HBV-related TCGA-HCC cohorts in TPX2^low^ and TPX2^high^ groups and discussed how this could affect the prognosis for HCC. The findings claimed a ceRNA regulation network to elucidate how TPX2 affects the prognosis of HBV-related HCC. Unless HCC is detected early and completely resected or abated, the prognosis is grave. Yin et al. focused their study on treating HBV-related HCC with traditional Chinese medicine (TCM). In this study, using target-driven reverse network pharmacology, authors investigated TCM’s therapeutic potential in treating HBV-related HCC. In order to understand the biological processes and pathways regulated targets, a network of 47 targets was established and a functional analysis was also conducted. Finally, they obtained a small library of chemical components and herbs against HBV-related HCC.

Using viruses to selectively destroy cancer cells is another established concept. With the approval of several oncolytic virus agents for clinical use, oncolytic viruses have shown promising anti-tumor efficacies, especially in combination with other traditional treatments. Li et al. focused on the characteristics of oncolytic viruses EV-A71 and their mechanisms in tumor treatment. They revealed the molecular mechanism by which EV-A71 infection are able to target glioma cells from the perspective of ABCD3, and also discovered the relationship with immune factor interactions in this process. These innovative virotherapy results strengthened virus-based targeted therapy, while at the same time broadened the idea of gene-modified oncolytic viruses.

In conclusion, this Research Topic addressed the mechanisms by which DNA tumor viruses operate on cells during transformation. Based on the findings in these papers, there are new therapeutic targets, new diagnostic tools, and strategies that need to be applied to clinical practice in order to cure tumors caused by oncogenic viruses.

## Author contributions

MH, BW and CW conceived of the study and its design. MH and CW wrote the manuscript. All authors read and approved the final manuscript.
